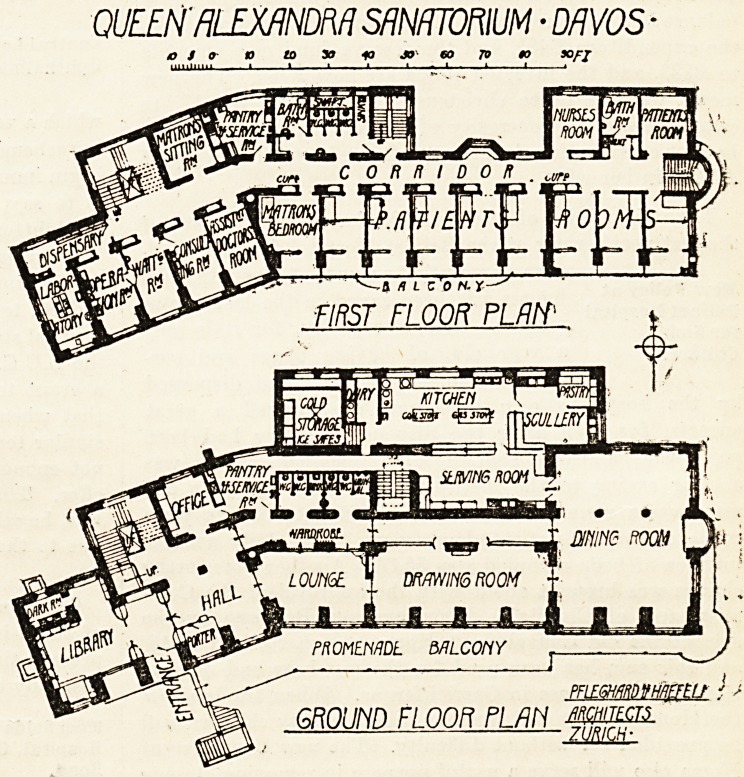# The Queen Alexandra Sanatorium, Davos Platz, Switzerland

**Published:** 1910-03-05

**Authors:** 


### THE QUEEN ALEXANDRA SANATORIUM, DAVOS PLATZ, SWITZERLAND.

This Sanatorium has been erected by voluntary contributions from all parts of the United Kingdom for the benefit of patients of small means suffering from tuberculosis. In the words of the founders, "It is designed to co-operate with, and to serve as an adjunct to, the various institutions in the United Kingdom which aim at the cure of patients suffering from tuberculosis and generally at checking the spread of that disease. In particular this Sanatorium is intended for the special benefit of the numerous classes midway between the well-do-do and the poor, who suffer great hardships from a protracted illness, but for whose benefit up to the present less provision has been made than for any other class of the community."

The building stands on the slopes of the Grüne Alp, about 300 feet above Davos, and as to its main block faces south, the west wing being slightly bent round to face about S.S.E. The situation is a magnificent one, and commands extensive views of the valley and the mountains. A pine forest extends down almost to the door and affords unlimited opportunities for exercise within easy reach of the patients.

Advantage has been taken of the steep incline on which the building stands to arrange two stories below the principal floor and to have two entrances at two different levels. The lowest floor of all, in which are placed the storage cellars, heating apparatus, disinfecting room and box-room, is partly below the ground, partly above. The next floor above, though called basement, is in reality a lower ground floor. It contains the servants' quarters, linen room, store room for dry goods, Röntgen ray room, entrance for goods with office, and the mortuary.

The ground floor is, therefore, for the greater part of its extent, well elevated above the ground in front, and only becomes what is commonly regarded as ground floor at the extreme west end. In the west wing is the entrance hall with a library immediately adjoining to the left. At the back is the main staircase, a dark room for photography, and office. The front part of the main building is occupied by a lounge, drawing-room and dining-room; the kitchen offices, with cold storage room, pantry, dairy, and service room, being at the back or north side. Two groups of sanitary offices open out of the corridor behind the lounge. The position of these offices, which is the same on the floors above, looks, to an eye accustomed to English sanitary arrangements, peculiarly open to criticism; but it is explained by the fact that owing to the severity of the climate in winter it is impossible to place the sanitary offices on an outside wall. A ventilating shaft is, therefore, constructed, as will be seen by referring to the first-floor plan, with which the sanitary offices on each floor communicate. This shaft is heated by steam-pipes to ensure a constant upward current of air. This arrangement, coupled with the fact that the sanitary fittings have been carried out by an English firm who have had previous experience of the conditions of the Swiss climate, should be sufficient guarantee of the efficiency of the precautions taken to avoid danger.

A wide balcony runs round from the entrance hall to the end of the drawing-room, and thence, reduced in width, along the south front of the dining-room and as far as the bow window on the east front.

The first floor contains nine rooms for patients, eight of which open on to a wide balcony, the matron's bedroom and her sitting-room, a nurses' room, two bathrooms, a pantry, and sanitary offices. At the west end is a room for the assistant medical officer, a consulting-room, waiting-room, operation-room, laboratory and dispensary. In recesses in the corridor are wardrobes for the use of the patients. It will be noted that the outer wall of each patient's room is nearly all window, the construction having been evidently carefully devised with that end in view. The four floors above are all planned in a similar way, except that patients' rooms take the place of the operation and adjoining rooms in the west wing.

The west wing is carried up a story higher to provide rooms for the medical superintendent and a board-room. A passenger lift in the well of the staircase in the west wing affords access to all floors.

The construction of the building is of fire-resisting materials throughout. The walls are built of stone quarried in the hill just behind the Sanatorium, which is covered with plaster and painted—the usual mode of construction in Davos. The floors and balconies are constructed of reinforced concrete, the floor surfaces inside the rooms being finished with cement and covered with linoleum. The staircases are formed of similar concrete.

The finishings inside are simple and plain, all walls and ceilings being finished with a smooth surface and all angles being rounded. The walls are covered to a height of seven feet with "Salubra," a material on linen which can be washed with disinfectants without damage; above this the walls and the ceilings are treated with Hall's washable distemper.

The building is heated throughout by low pressure steam, a large English fireplace being provided in the lounge, which must be a real boon to patients accustomed to our method of warming. The lighting throughout is electric, and electric bells and local telephones are provided.

Disinfection of rooms is carried out by the Communal Authorities by means of formalin vapour. After this has been done the rooms are thoroughly washed out with perchloride of mercury (1 in 1,000) by the Sanatorium staff. At present the institution possesses no disinfecting apparatus for clothes or bedding, and all things requiring disinfection are sent to the Davos disinfecting station, which is under the control of the Public Health Department. Sputum flasks, etc., are sterilised by high pressure steam.

The accommodation for patients in the building as it stands to-day is 54 beds; but the administrative offices have all been planned with a view to an increase of 50 beds should the demand justify the Board in making such addition. The plan is simple but admirably adapted to the purpose, and the treatment of the exterior, though severely plain, possesses a certain dignity which results from the skilful handling of large masses without recourse to meretricious ornament; moreover, it expresses the object and use of the building beyond the possibility of doubt, which is the proper aim of good architecture.

The architects are Messrs. Pfleghard and Haefeli, of Zurich, who are to be congratulated on the completion of their excellent work.

## Figures and Tables

**Figure f1:**